# Origin and haplotype diversity of the northernmost population of *Podarcistauricus* (Squamata, Lacertidae): Do lizards respond to climate change and go north?

**DOI:** 10.3897/BDJ.10.e82156

**Published:** 2022-04-18

**Authors:** Ivan Rehák, David Fischer, Lukáš Kratochvíl, Michail Rovatsos

**Affiliations:** 1 Prague Zoo, Prague, Czech Republic Prague Zoo Prague Czech Republic; 2 Mining Muzeum Příbram, Příbram, Czech Republic Mining Muzeum Příbram Příbram Czech Republic; 3 Department of Ecology, Faculty of Science, Charles University, Prague, Czech Republic Department of Ecology, Faculty of Science, Charles University Prague Czech Republic

**Keywords:** *Podarcistauricus*, wall lizards, autochthonous population, introduction, Czech Republic, new haplotypes, *cytb*

## Abstract

The northernmost population of the Balkan wall lizards, *Podarcistauricus* (Pallas, 1814) was recently discovered in the Czech Republic. We studied genetic variability in a mitochondrial marker *cytochrome b* to shed light on the origin of this remote population. We detected three unique haplotypes, close to those occurring in the populations of *Podarcistauricus* from central/north Balkans and Hungary. Our data exclude the hypothesis of a single founder (a randomly or intentionally introduced pregnant female or her progeny) of the Czech population and indicate a native, autochthonous origin of the population or recent introduction/range expansion.

## Introduction

The Balkan wall lizard (also referred as the Crimean wall lizard), *Podarcistauricus* (Pallas, 1814) is a small, diurnal, heliothermic and actively foraging lizard. Being a ground-dwelling lizard adapted to open sandy steppe habitats with sparse vegetation, it differs ecologically from most of the other wall lizards of the genus *Podarcis* Wagler, 1930. Currently, according to Psonis et al. (2017) who synonymized *P.t.thasopulae* (Kattinger, 1942) with *P.t.tauricus* and separated *P.t.ionicus* (Lehrs, 1902) as a distinct Albanian-Greek species complex, *Podarcistauricus* is considered to be a monotypic species. For additional information on the Balkan wall lizard’s phylogeography, see also Chondropoulos et al. (2000), Oliverio et al. (2000), Poulakakis et al. (2005) and Psonis et al. (2018).

**Figure 1.**
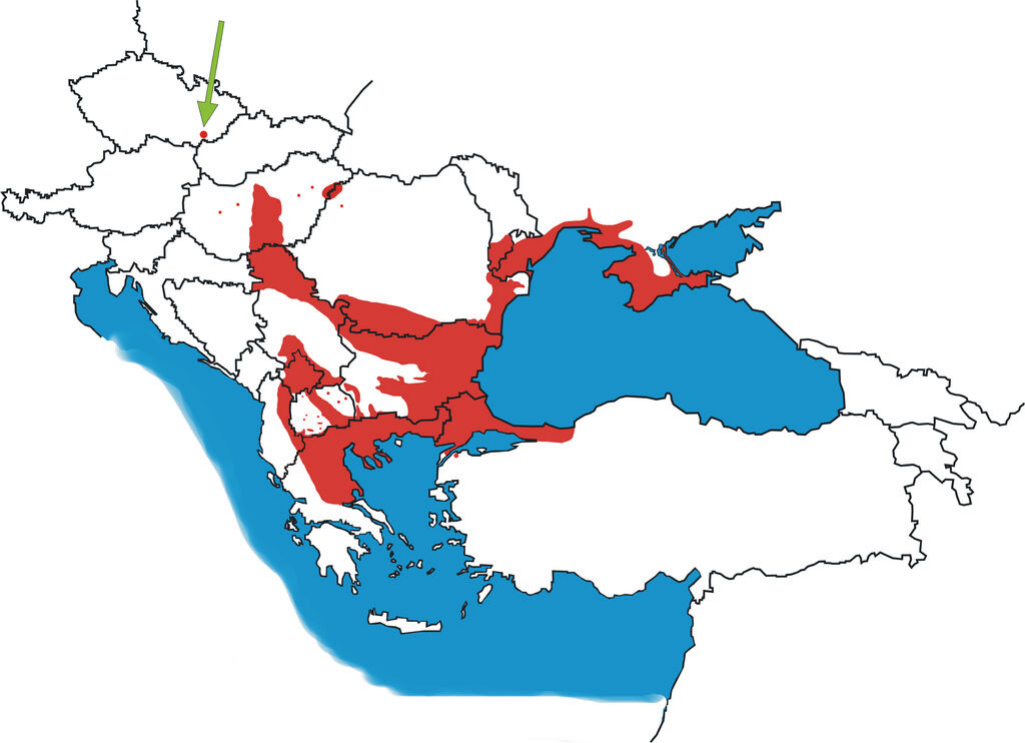
Geographical distribution of *Podarcistauricus*. The green arrow shows the northernmost known locality ("Váté písky", Czech Republic). Data are collected from Dely and Kovács (1961), Dely (1965), Bannikov et al. (1977), Mulder (1995), Sindaco et al. (2000), Petrov (2007), Tzankov (2007), Böhme et al. (2009), Ljubisavljević et al. (2010), Cogălniceanu et al. (2013), Sillero et al. (2014), Tomović et al. (2014), Bülbül et al. (2015), Uhrin et al. (2016), Gül and Tosunoğlu (2017), Psonis et al. (2017), Psonis et al. (2018), Çördük et al. (2018), Fischer et al. (2019), Oskyrko and Jablonski (2021), Hungarian Ornithological and Nature Conservation Association (https://www.mme.hu), National Red List of North Macedonia (http://redlist.moepp.gov.mk), Ukrainian Biodiversity Information Network (http://www.ukrbin.com), GBIF (http://www.gbif.org) and iNaturalist (http://www.inaturalist.org).

The geographical distribution of the species is wide and, in addition to areas with a continuous range, it also includes isolated local populations (Fig. 1). The northwest borders of the species continuous range are in Hungary, where *P.tauricus* is distributed predominantly east of the Danube River in lowlands between the Danube and the Tisza. In addition, there are geographically isolated populations in Hungary and north-western Romania.

Štěpánek (1949), Lác (1968) and Ponec (1978) did not exclude distribution of *P.tauricus* in Slovakia (e.g. the Burda and Slovak Karst). Rozínek et al. (1974) reported this species from Vihorlat Mountains in eastern Slovakia, but photographic evidence or voucher specimens were not provided. Rehák (1992) pointed out that *Podarcistauricus* has never been credibly documented from former Czechoslovakia and that its occurrence would be more likely expected in the lowlands. He concluded that the present range of *P.tauricus* apparently does not reach the Slovak borders. Notably, *Podarcistauricus* is not mentioned in the recent monograph on reptiles of the Czech Republic (Moravec 2015). However, to our great surprise, a viable population of *Podarcistauricus* has recently been discovered near Bzenec, South Moravia, Czech Republic (Fischer et al. 2019), more than 200 km away from the nearest known locality in Hungary. The origin of this remote population remains unclear. Fischer et al. (2019) provided the data on morphology, colouration, ecology and ethology of the local lizards, taxonomically assigned as *Podarcistauricus*, but did not study any genetic aspects. In the present study, we focused on the haplotype diversity of this unique population to obtain insight into its phylogenetic relationships to other populations of *P.tauricus* and its possible origin.

## Material and methods

**Figure 2.**
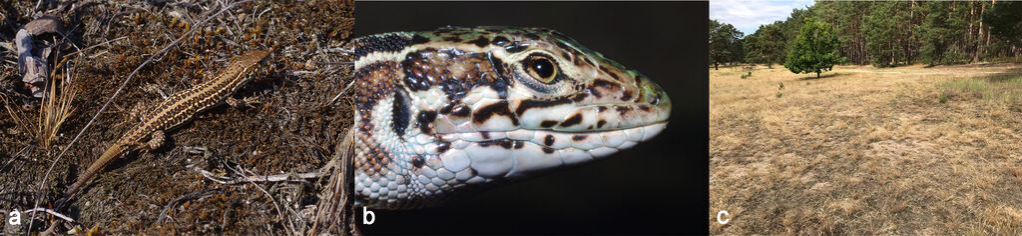
Photographs of *Podarcistauricus*, whole specimen (a), portrait (b) and its habitat (c) from "Váté písky" near Bzenec, the only known locality of this species in the Czech Republic.

We collected tail tips from seven specimens of *Podarcistauricus* from the locality "Váté písky" near Bzenec in the South Moravian Region, Czech Republic, during a fieldtrip in 2019 (Figs 1, 2). Tail tips were preserved in ethanol and the lizards were released within their home range. Total DNA was isolated by the DNeasy Blood and Tissue Kit (Qiagen), using the manufacturer’s protocol. The partial *cytochrome b* (*cytb*) locus was amplified by PCR with the primers L14919 5’-AACCACCGTTGTTATTCAACT-3’ (de Queiroz et al. 2002) and H16064 5’-CTTTGGTTTACAAGAACAATGCTTTA-3’ (Burbrink et al. 2000). Our detailed protocols for cycler conditions and chemistry were previously published in Mazzoleni et al. (2019). The PCR products were sequenced bi-directionally in Macrogen, Korea. The sequences were trimmed in Geneious Prime (www.geneious.com) and compared to previously deposited datasets in the GenBank database by the Basic Local Alignment Search Tool (https://blast.ncbi.nlm.nih.gov/Blast.cgi). Haplotypes were extracted by DnaSP v.6 (Rozas et al. 2017), pulled together with 160 sequences *Podarcistauricus* and *Podarcisgaigeae* reported in Psonis et al. (2017), and a haplotype network was reconstructed by Haploviewer (Barrett et al. 2004). A phylogenetic tree of haplotypes, needed for Haploviewer, was prepared by the Maximum Likelihood algorithm in MEGA v.10 (Kumar et al. 2018). The haplotypes of our analyzed wall lizards are deposited in GenBank, under the accession numbers ON155608-ON155610. All methods were carried out in accordance with relevant Czech and EU guidelines and regulations (permit numbers MSMT-35484/2015-14 and UKPRF/28830/2021).

## Results

**Figure 3.**
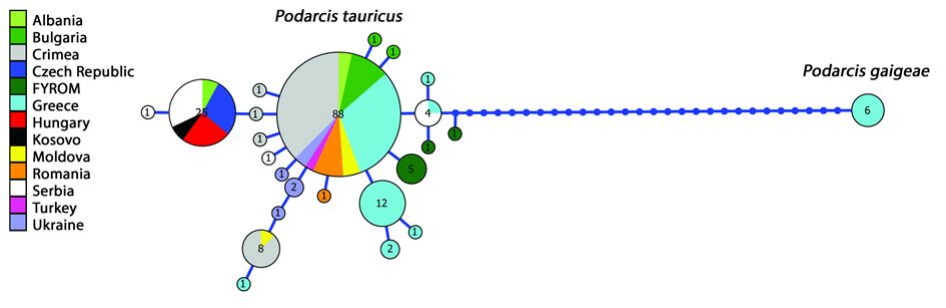
Haplotype network, designed from 24 haplotypes of the *cytb* locus from 167 individuals of *Podarcistauricus* and *Podarcisgaigeae* (Psonis et al. 2017; this study). Colours correspond to the country of the specimen’s geographical origin and each circle corresponds to a haplotype. The circle size is proportional to the number of individuals with the same haplotype. The number of individuals per haplotype is indicated. Due to the unequal size of *cytb* sequences from Psonis et al. (2017), only a fragment of 257 bp which was common for all 167 sequences was used for the haplotype network reconstruction. For this region of *cytb* locus, the sequences of our individuals from Czech Republic are identical to 18 individuals from Albania, Hungary, Kosovo and Serbia.

We have obtained the partial sequence of the *cytb* locus (1124 bp) from seven specimens of *Podarcistauricus*. We detected three different haplotypes (1124 bp sequence length, haplotypes differ in three sites). All of them are unique, first reported, novel haplotypes of *Podarcistauricus* (cf. Psonis et al. 2017, Psonis et al. 2018) (alignment of 257 bp, 98.44-100% similarity, 0-3 mismatches with *cytb* sequences from Psonis et al. 2017). The *cytb* sequences from the individuals from the Czech Republic are very close to *cytb* sequences from individuals from Albania, Hungary, Kosovo and Serbia (alignment of 257 bp, 100% similarity with *cytb* sequences from Psonis et al. 2017). We can conclude that the studied population from Czech Republic is phylogenetically closely related to these populations (Fig. 3).

Our data indicate that the population from "Váté písky", being the northernmost population of the species, could be native, autochthonous, representing either: (i) isolated population derived from the expansion/migration of *P.tauricus* from central/northern Balkans or Hungary, for example from post-glacial colonisation or (ii) remnant, isolated sub-population of a previously (perhaps even prior to the Last Glacial Maximum) widespread distribution across central/north Balkans, Hungary and parts of the Czech Republic. At the same time, our data do not exclude range expansion or human-induced transfer of individuals from southern populations, including the possibility that *P.tauricus* has been recently introduced to the Czech Republic by humans from a genetically unstudied population of central/northern Balkans or Hungary. However, our data clearly exclude an origin of the population from a single, randomly or intentionally introduced pregnant female or her progeny.

## Discussion

Our results provide two basic alternative explanations for the origin of the *P.tauricus* population in the Czech Republic: natural occurrence or direct, human-induced introduction.

The first hypothesis assumes a natural occurrence of the Czech population of *P.tauricus*, either due to a relatively recent range expansion or being a relict of an older continuous distribution. This scenario could be supported by several lines of evidence:

(1) It agrees with the detection of three, unique, previously unsampled haplotypes amongst our seven examined individuals, phylogenetically related to haplotypes from central/north Balkans and Hungary. Such genetic variability makes an unintentional random introduction less likely.

(2) Historically, there were large areas of eolian sands in the Dyje, Morava and Danube Basins, i.e. between the locality of *P.tauricus* in South Moravia and the distribution in Hungary. These areas apparently provided suitable habitats for the lizard and, thus, opportunities for migration corridors, as well as an ancient distribution.

(3) Railway embankments, artificially maintained without bushes and higher vegetation could very well serve as a possible corridor for eventually recent spread from areas of autochthonous occurrence. Such embankments are generally widely used by reptiles and, in this case, the railway route from Bzenec to the localities of *Podarcistauricus* in Hungary can be easily traced.

(4) According to our data, *P.tauricus* has low abundance and is syntopic with the European green lizard, *Lacertaviridis* (Laurenti, 1768) at the locality "Váté písky". Subadults of *L.viridis* may be surprisingly similar to *P.tauricus* and even field herpetologists, misled by the paradigm that only *L.viridis* occurs in this area, can very easily overlook the existence of *P.tauricus*, which is harder to spot because it is very alert and has highly developed escape behaviour.

The alternative hypothesis suggests a recent, human-induced, intentional or unintentional introduction of the Balkan wall lizard to the Czech Republic from Hungarian or Balkan populations. This scenario could be supported by the following facts:

(1) According to our data, the current population is limited to a very small area. Despite our intensive field research, we have not yet been able to confirm the occurrence of *P.tauricus* elsewhere in the Czech Republic. Nevertheless, according to our latest observations from 2021, we can confirm that the existing population is still viable and reproducing.

(2) The Czech Republic and Slovakia are relatively well researched in terms of vertebrate occurrence. The probability that a lizard species has remained overlooked for so long is low, which would indicate that the newly-discovered population was established very recently.

(3) The location is close to the railway line with very intensive operation of train sets from Hungary and the Balkans, transporting both passengers and cargo. Nearby, there are intensively used sand quarries, whose activities are associated with long-distance transport of sand, as well as machinery and materials. In addition, there is intense road traffic in the area. These conditions increase the possibility of unintentional introduction of *P.tauricus*, which could have occurred more than once, justifying the increased genetic variability of the Czech population. Several unintentional introductions of lizards to the Czech Republic were previously documented. For example, the Italian wall lizard, *Podarcissiculus* (Rafinesque-Schmaltz, 1810) was found inside a consignment of vegetables in Prague Zoo (Velenský 2019) or another wall lizard climbed out of a cactus purchased in a local gardening store (Kuba O, 6.1.2022, in litt.) and, in a third independent case, a wall lizard was unintentionally imported from the Island of Vrgada, Croatia to the town of Krušovice, inside a tourist backpack (Fischer L, July 2019, in verb).

(4) Attempts to deliberately introduce a non-native species were documented in the Bzenec area. For example, a small non-native population of the locust *Acridaungarica* (Herbst, 1786) was discovered here in 2010, which survived but did not tend to spread. Genetic analyses have shown that the introduced population belongs to southern European subspecies *A.ungaricamediterranea* Dirsh, 1949 and not to the nominotypic subspecies present at the nearest southern Slovak populations (Vlk et al. 2014, Koláčková 2017). We cannot rule out that individuals of *P.tauricus* were intentionally released in the Bzenec area by humans, because, in this case, the genetic analysis could not distinguish between a local, natural origin and an artificial introduction from a proximate natural population. A similar situation can be found in the Common wall lizard, *Podarcismuralis* (Laurenti, 1768), whose local populations in the Czech Republic (and similarly in Poland and some populations in Ukraine) may have arisen as a result of intentional or unintentional human-mediated introduction. The comparison of haplotypes alone cannot unambiguously determine whether the Common wall lizard is introduced or autochthonous (Jablonski et al. 2019, Vlček and Zavadil 2019, Kolenda et al. 2020, Oskyrko et al. 2020).

In the Czech Republic, we have several cases in which reptile species have been intentionally introduced by humans in a number sufficient to establish a viable population. Between 1989 and 1994, a total of 30 (9 males, 21 females) European Pond Turtles, *Emysorbicularis* (Linnaeus, 1758), originally from the Romanian part of the Danube Delta, were intentionally released by reptile enthusiast(s) into the protected wetlands of Betlém, South Moravia and established a prosperous and expanding local population (Šebela 2017). In addition, there are strong indications that the viable and reproductive population of the Aesculapian snake, *Zamenislongissimus* (Laurenti, 1768) in the middle Vltava River valley in Central Bohemia, which we are currently monitoring for the Nature Conservation Agency of the Czech Republic, is also the result of introductions from other parts of its distribution area during the 1970s and 1980s by reptile enthusiast(s) (own data). As a very current (discovered in 2021), probably intentional introduction, we can mention the occurrence of the Common wall lizard, *Podarcismuralis*, directly in Prague (National Monument at Vítkov; 15 observed individuals, reported by Jan Kubát on 3.10.2021 and evidenced by photos at www.biolib.cz). An example of a large number of potential founders to establish a new population is the finding of 264 tortoises *Testudohermanni* GMELIN, 1789, a species native to southern Europe, which were deliberately released in a single locality in the outskirts of Prague in 2017. The owner(s) of the turtles and the motives behind this release remain unknown; the turtles were collected by the authorities and transferred to Prague Zoo and Plzen Zoo, where we performed their taxonomical identification, based on morphological and molecular markers.

Although we cannot decisively conclude on the natural occurrence or the direct human-induced introduction of *Podarcistauricus* in Czech Republic, we will continue to monitor the population. Notably, we will focus on assessing if the ongoing global warming, which gradually raises the average temperature in the wider region (see data from Zahradníček et al. 2020), will create favourable conditions for the survival of the *P.tauricus* population in the Bzenec area and will drive its further geographical expansion. Our study significantly contributes to the understanding of the haplotype diversity, phylogenetic relationships and the origin of the single population of *P.tauricus* in the Czech Republic; however, further research is needed to determine its origin more accurately. In any case, regardless of the origin of this population, it remains exceptionally interesting and should deserve conservation actions as potentially autochthonous.
